# Fellowships, Grants, & Awards

**Published:** 2006-05

## Pilot and Feasibility Clinical Research Grants in Kidney or Urologic Diseases (R21)

The Division of Kidney, Urologic, and Hematologic Diseases of the National Institute of Diabetes and Digestive and Kidney Diseases (NIDDK) has a longstanding and substantial interest in research concerning the prevention and treatment of kidney or urologic disorders. This program announcement is a reissuance of PAR-04-065, and specifically encourages the submission of applications for pilot and feasibility clinical and translational research studies, including clinical trials, and epidemiologic studies, related to kidney or urologic disease research that address important clinical and translational questions and are potentially of high impact. It is anticipated that applications for pilot and feasibility studies may lead to full-scale clinical studies, including diagnostic strategies, epidemiologic studies, or trials in the diagnosis, prevention, preemption, or treatment of kidney or urologic disease.

These grants may be used for plan, pilot, or implement trials that evaluate pharmacologic, dietary, surgical, or behavioral interventions for the prevention or treatment of kidney or urologic disease. Pilot epidemiologic studies are also encouraged. It is anticipated that these grants will in some cases serve as a basis of planning future multicenter research project grant applications (R01), or for cooperative agreement (U01) applications. The purpose of the planning grant is to provide support for investigators at different institutions to meet and design common protocols, entry criteria, data management systems, analysis plans and pilot data for a clinical trial. This would allow investigators to obtain additional expertise during the trial planning phase in areas such as clinical trial design and statistics to develop an R01 grant to support the clinical trial. A pilot clinical grant to plan a large clinical trial (defined as a trial projected to exceed $500,000 direct costs per year) will only be accepted after prior discussions and approval from NIDDK staff. Both new and experienced investigators in relevant fields and disciplines are encouraged to apply for these grants.

It is anticipated that applications submitted in response to this PAR will focus on clinical studies. Basic laboratory research, studies of laboratory animals, or clinical hematology studies are not appropriate for this program announcement. Studies that do not involve human subjects or are not human studies will not be supported through this funding opportunity. Applications that focus on experimental models of disease are not appropriate for this PAR, but should be submitted to PA-05-103.

Recent estimates of chronic kidney disease (CKD) in the U.S. population, obtained through analysis of the Third National Health and Nutrition Examination Survey (NHANES III), indicate that it is a common medical problem, affecting ≥10 million people in the U.S. population. Most cases of CKD observed in the United States occur in the setting of diabetes, hypertension, glomerulonephritis, and polycystic kidney disease. The incidence of end-stage renal disease (ESRD) has also been steadily increasing in the adult US population. U.S. Renal Data System (USRDS) data indicate that from 1992–2003 the number of patients with ESRD has increased from 250 to 338 per million population. Although these rates are finally stabilizing, these increases in ESRD rates reflect a marked increase in patient morbidity and mortality related to underlying kidney disease, as well as a significant increase in use of health care resources to provide appropriate care for affected patients. The increasing rate of ESRD has also markedly increased waiting time for cadaveric transplantation such that the rate of kidney transplants per patient year on dialysis has steadily declined over the last decade, from 6.7 per 100 dialysis patients in 1991 to 4.7 in 2003.

Acute kidney injury (AKI; also called acute renal failure) in hospitalized patients is also a significant problem in the United States. Medical management of acute kidney injury has traditionally consisted of supportive care, with renal replacement therapy implemented for the most severe cases. Despite such interventions in acute kidney injury, however, mortality rates in affected patients remain very high (> 50% in some series).

In view of these observations suggesting a high prevalence of CKD, and increasing ESRD and AKI in the U.S. population, NIDDK has sponsored a number of large, multicenter studies of specific kidney disorders. These studies include prospective investigations in chronic kidney disease, dialysis access, polycystic kidney disease, focal and segmental glomerulosclerosis, and acute kidney injury. In planning and performing these studies, however, it has been apparent that the process for identifying appropriate interventions for both single and multicenter trials in kidney disease could be improved. This is particularly evident in the current small number of clinical studies related to kidney disease that could ultimately be expanded to large-scale clinical trials.

Urological diseases and disorders inflict a significant impact on the health care burden of the United States. The NIDDK-funded Urologic Diseases in America Project (UDA) has published data on the four of the most prevalent nonmalignant urological diseases. The data indicate that in 2000, benign prostatic hyperplasia was the primary diagnosis in > 4.4 million office visits, 424,000 emergency room visits and 121,000 hospitalizations with an annual expenditure of > $1.1 billion, besides the increasing cost for outpatient pharmaceuticals. The annual expenditures for urolithiasis totaled more that $2 billion and appears to be increasing with time as the prevalence of stone disease also increases. The health care burden for urinary tract infections, excluding the costs of outpatient pharmaceuticals, exceeded $2.47 billion.

The data for the health care burden of urinary incontinence are more difficult to accurately ascertain because a large percentage of women and men do not report or access care for urinary incontinence. However, the UDA reported that medical expenditures and outpatient visits for urinary incontinence more than doubled from 1992 to 1998. The data for other nonmalignant urological diseases and disorders such as erectile dysfunction, the chronic pelvic pain syndromes such as chronic prostatitis and interstitial cystitis, and the many congenital and acquired pediatric urological disorders are not as rigorous but still demonstrate large and growing health care burden.

The morbidity of many of these nonmalignant urological disorders is compounded by other common comorbid conditions such as diabetes and obesity. Advancement in accurate diagnosis, prevention, and treatment of these diseases is hampered by many factors including a lack of vigorously validated methods to access disease progression, by a lack of insight into the genetics of the disorders, by well-formulated and-tested definitions of the disease and its subcategories, by a lack of rigorous epidemiologic data, and by an imperfect understanding of the mechanism of action of the drugs used to alleviate the symptoms of these diseases and disorders. In addition, often the armamentarium of available diagnostic approaches has not been applied to these diseases.

The goal of this PAR is to provide flexibility for initiating preliminary, short-term studies, thus allowing new ideas to be investigated in a more expeditious manner without stringent requirements for preliminary data. Such support is needed to encourage new and experienced investigators to pursue new approaches, underdeveloped topics, or more risky avenues of research. If successful, these awards should lead to significant scientific advances in the treatment of kidney diseases.

As the kidney and urological diseases occur in a variety of clinical settings, and are associated with a number of comorbid conditions, applications submitted in response to this PAR could address a number of different aspects concerning the prevention, diagnosis, or treatment of patients with kidney and urological diseases.

Relevant topics of study evaluating kidney disease in adults or children could include, but are not limited to 1) diagnosis, epidemiology, disease progression, prevention, preemption, or therapy of patients with, or at risk for, the following conditions: chronic kidney disease, including studies of diabetic nephropathy; hypertensive nephrosclerosis, polycystic kidney disease, or renal allograft dysfunction; glomerular disease, either idiopathic or secondary glomerular involvement in a systemic process; acute kidney injury, including that observed following renal transplantation; comorbid conditions associated with reduced kidney function; 2) studies assessing dialysis therapy, dialysis access, anemia of renal disease, nutritional, or cardiovascular aspects of ESRD and other comorbid conditions associated with ESRD.

Relevant topics for study of the nonmalignant urological diseases in adults or children could include, but are not limited to 1) diagnostic tools and instruments that can asses the extent and physiological parameters of disease and evaluate disease progression or response to therapy; 2) improved diagnostic criteria for diseases and disease subcategories; 3) accurate epidemiologic data on diseases in various ethnic and racial groups; 4) validated strategies to access early detection of disease, for disease progression and for response to therapy; 5) novel approaches to preventing the onset of disease or preventing the progression of established disease; 6) studies of the effect of the treatment of comorbid disorders on the symptoms, progression, and morbidity of urological diseases.

This funding opportunity will use the NIH Exploratory/Development Research Grant (R21) award mechanism. Information on R21 mechanism is available at http://grants.nih.gov/grants/guide/pa-files/PA-03-107.html. These R21 grants will not be renewable; continuation of projects developed under this program will be through the regular research grant (R01) program as new applications. As an applicant, you will be solely responsible for planning, directing, and executing the proposed project.

This funding opportunity uses just-in-time concepts. It also uses the modular as well as the nonmodular budget formats (see http://grants.nih.gov/grants/funding/modular/modular.htm). Specifically, if you are submitting an application with direct costs in each year of $250,000 or less, use the modular budget format described in the PHS 398 application instructions. Otherwise follow the instructions for nonmodular research grant applications.

The PHS 398 application instructions are available at http://grants.nih.gov/grants/funding/phs398/phs398.html in an interactive format. Applicants must use the currently approved version of the PHS 398. For further assistance contact Grants Info, 301-435-0714 (telecommunications for the hearing impaired: TTY 301-451-0088) or by e-mail: GrantsInfo@nih.gov.

Applications must be prepared using the most current PHS 398 research grant application instructions and forms. Applications must have a D&B Data Universal Numbering System (DUNS) number as the universal identifier when applying for Federal grants or cooperative agreements. The D&B number can be obtained by calling 866-705-5711 or through the web site at http://www.dnb.com/us/. The D&B number should be entered on line 11 of the face page of the PHS 398 form.

The application submission dates for this PA are available at http://grants.nih.gov/grants/funding/submissionschedule.htm. The complete version of this PA is available at http://grants/nih.gov/grants/guide/pa-files/PAR-06-112

Contacts: Robert A. Star, Division of Kidney, Urologic and Hematologic Diseases, National Institute of Diabetes and Digestive and Kidney Diseases, 6707 Democracy Blvd., Room 612, Bethesda, MD 20892-5458 USA, 301-594-7717, fax: 301-480-3510, e-mail: rs301p@nih.gov; Catherine Meyers, Division of Kidney, Urologic and Hematologic Diseases, National Institute of Diabetes and Digestive and Kidney Diseases, 6707 Democracy Boulevard, Room 725, Bethesda, MD 20892-5458 USA, 301-594-7717, fax: 301-480-3510, e-mail: cm420i@nih.gov.

Reference: PAR-06-112

## Autism Centers of Excellence (R01)

Autism spectrum disorders (ASD) are complex neurodevelopmental disorders with early childhood onset. ASD prevalence may be increasing, and ASD is more common than previously thought. These disorders, for which there is presently no cure and only limited treatments, generally have lifelong effects.

The NIH currently supports a vast array of projects in autism research. Centers such as the STAART (Studies to Advance Autism Research and Treatment) and CPEA (Collaborative Programs of Excellence in Autism) programs support some of these investigations. The CPEA program, an international program begun in 1997, now includes nine centers. These Centers focus research on the possible causes of autism, including genetic, immunological, and environmental factors. The CPEA program resulted from a congressionally mandated conference on the State of the Science in Autism. The attendees identified gaps in the knowledge of autism and directions for future research. Both NICHD and NIDCD sponsor the current CPEA program. As a result of the efforts of researchers affiliated with the CPEA, data now exist on the genetics and outward characteristics of the largest group of well-diagnosed persons with autism in the world. After the establishment of the CPEA Centers program, Congress enacted the Children's Health Act of 2000. This legislation mandated the establishment of a new autism research program. In response, the five Institutes of the NIH Autism Coordinating Committee (NIH-ACC; represented by NICHD, NIDCD, NIEHS, NIMH, and NINDS) implemented the STAART program. Each of the eight currently funded STAART Centers contributes to the autism research base in the areas of causes, diagnosis, early detection, prevention, and treatment. Collaborations among the STAART centers include a multisite psychopharmacological clinical trial. The CPEA and the STAART programs interact extensively through a shared data coordination center.

Consolidation of funding from the CPEA and STAART programs is now needed to maximize coordination and cohesion of NIH-sponsored efforts in autism research. The new autism centers and networks will be called the Autism Centers of Excellence or ACE.

The focus of the ACE must be on the causes and best treatment of autism (as listed in the autism research matrix http://www.nimh.nih.gov/autismiacc/research-matrix.pdf). In February 2003, Congress requested that the Department of Health and Human Services (DHHS) develop a set of autism research goals and activities for the next several years (House Report 109-10). Input into this activity included a meeting of autism investigators with a range of scientific expertise as well as input from community members. Preparation for specifying this matrix involved a two-day meeting of an expert panel of scientists; public presentation and discussion of a draft matrix at the Autism Summit Conference in Washington DC on 20 Novembwe 2003; and adoption of the matrix by the Federal Interagency Autism Coordinating Committee (IACC).

Research projects should focus on finding causes and preventive intervention for autism as well as improved treatment. Applications should incorporate the latest techniques and propose studies to advance key goals related to causes and best treatment on the autism research matrix. Examples include, but are not limited to 1) identification of individual characteristics that predict response to behavioral, pharmacologic, and other treatments; 2) identification of environmental factors (e.g., viruses, medication, lifestyle factors, environmental chemicals) that contribute to the development of autism and their associated developmental windows; 3) identification of the biologic and/or behavioral markers to develop indices of risk for the development of autism in infants; 4) intervention methods for infants and toddlers developed to lower the age for which there are efficacious interventions; 5) multisite randomized clinical trials to identify moderators and effective ingredients (e.g., dose, intensity, mode of delivery, age of onset) of early intervention treatments; 6) multisite longitudinal study of subsequent pregnancies and infant siblings of children with autism to identify risk factors, broader phenotype, and early characterization of autism; 7) innovative and newly developed intervention strategies to improve outcomes in school and community settings throughout the lifespan (e.g., academic functioning, social and adaptive behavior, family functioning, employment), including transitions; 8) development of efficacious drug treatments that target core symptoms of autism; 9) characterization of the neuropathology of autism to identify impaired brain structures and functions; 10) identification of susceptibility genes and animal models of autism for further study of phenotypic characteristics of autism.

NIH will consider centers as well as networks in response to the ACE initiative. This funding opportunity solicits applications for ACE Networks supported by the R01 mechanism. A companion RFA (RFA-HD-06-016) solicits applications for ACE Centers supported by the P50 mechanism.

This funding opportunity solicits applications for ACE Networks. A network will consist of multiple sites focusing on a specific topic of research for R01 support through this funding opportunity. Each network will submit one R01 application that includes subcontracts to the collaborating sites. An ACE Network application must include one or more collaborative projects that require multiple sites for optimal design and conduct of studies. For example, an interrelated series of clinical trials of pharmacologic, behavioral interventions, or a combination of these, could be an example of the focus of such a network. Other examples include, but are not limited to, a multisite network that could conduct one or more neuroimaging protocols and collect data using common standards and a multisite epidemiology study of risk factors (e.g., genetic, environmental) for ASD. Special populations requiring large numbers of participants for each protocol may also be studied best under a network because of enhanced recruitment and other benefits of multisite subject accrual.

A companion RFA solicits applications for ACE Centers (RFA-HD-06-016). Centers bring together expertise, infrastructure, and resources focused on major questions about autism. Centers should involve collaborations of basic and clinical scientists optimally suited to address the research questions posed. NIH expects Centers to provide an environment and core resources to bring together biomedical, behavioral, and clinical science investigators to study autism. Collaborations involving more than one institution are strongly encouraged to provide optimal resources and expertise. The centers should provide investigators with well-characterized patients and control subjects, family information, and other scientific resources that facilitate research projects. Applications for centers must include a minimum of three but no more than six research projects. The addition of core support is optional and will depend on specific needs.

An ACE Center must support major multidisciplinary research programs, consisting of interdependent and interrelated subprojects. Meaningful and committed interactions among the disciplines must be evident. Subprojects may share materials, results, data, patient populations, or methodologies. Results of one subproject may well affect the understanding and interpretation of data from another project and thereby influence the nature of the research performed in one or more of the other subprojects. In addition, each subproject must have goals and objectives that focus on the common unifying theme that interrelates the subprojects.

NIH is developing a National Database for Autism Research (NDAR) to facilitate data sharing. NDAR (http://ndar.nih.gov) is both a database and a set of tools for autism researchers—a national resource for collaboration in autism research and clinical practice.

All ACE Centers and Networks will be required to contribute data to NDAR. Data sharing is required and NDAR will be involved to facilitate sharing activities. NDAR will function as a data repository for all ACE projects. Central clinical coordination and local data management for data cleaning and entry and biostatistical consulting will be the responsibility of the ACE Center or Network. Depending on the number and size of the multsite projects, NDAR may provide Data Coordinating Center (DCC) functions for large ACE multisite projects to increase efficiencies.

This funding opportunity will use the Traditional Research Project Grant (R01) award mechanism. As an applicant, you will be solely responsible for planning, directing, and executing the proposed project.

This funding opportunity uses just-in-time concepts. It also uses the modular as well as the nonmodular budget formats (see http://grants.nih.gov/grants/funding/modular/modular.htm). Specifically, if you are submitting an application with direct costs in each year of $250,000 or less, use the modular budget format described in the PHS 398 application instructions. Otherwise follow the instructions for nonmodular research grant applications.

The PHS 398 application instructions are available at http://grants.nih.gov/grants/funding/phs398/phs398.html in an interactive format. Applicants must use the currently approved version of the PHS 398. For further assistance contact GrantsInfo, 301-435-0714 (telecommunications for the hearing impaired: TTY 301-451-0088) or by e-mail: GrantsInfo@nih.gov.

Applications must be prepared using the most current PHS 398 research grant application instructions and forms. Applications must have a D&B Data Universal Numbering System (DUNS) number as the universal identifier when applying for Federal grants or cooperative agreements. The D&B number can be obtained by calling 866-705-5711 or through the web site at http://www.dnb.com/us/. The D&B number should be entered on line 11 of the face page of the PHS 398 form.

The letter of intent receipt date for this RFA is July 11, 2006, with the application receipt date August 11, 2006. The complete version of the RFA is available at http://grants.nih.gov/grants/guide/rfa-files/RFA-HD-06-004.html.

Contact: Alice Kau, Mental Retardation and Developmental Disabilities Branch, Center for Developmental Biology and Perinatal Medicine, National Institute of Child Health and Human Development, 6100 Executive Boulevard, Room 4B09F, MSC 7510, Bethesda, MD 20892-7510 USA, Rockville, MD 20852 USA (for express/courier service; non-USPS service), 301-496-1383, fax: 301-496-3791, e-mail: kaua@mail.nih.gov.

Reference RFA-HD-06-004

